# Efficient and accurate identification of maize rust disease using deep learning model

**DOI:** 10.3389/fpls.2024.1490026

**Published:** 2025-02-06

**Authors:** Pei Wang, Jiajia Tan, Yuheng Yang, Tong Zhang, Pengxin Wu, Xinglong Tang, Hui Li, Xiongkui He, Xinping Chen

**Affiliations:** ^1^ Key Laboratory of Agricultural Equipment for Hilly and Mountain Areas, College of Engineering and Technology, Southwest University, Chongqing, China; ^2^ Interdisciplinary Research Center for Agriculture Green Development in Yangtze River Basin College of Resources and Environment, Southwest University, Chongqing, China; ^3^ College of Plant Protection, Southwest University, Chongqing, China; ^4^ Chongqing Academy of Agricultural Sciences, Institute of Agricultural Machinery, Chongqing, China; ^5^ Centre for Chemicals Application Technology, College of Science, China Agricultural University, Beijing, China

**Keywords:** maize, southern rust, common rust, SimAM, small target detection

## Abstract

Common corn rust and southern corn rust, two typical maize diseases during growth stages, require accurate differentiation to understand their occurrence patterns and pathogenic risks. To address this, a specialized Maize-Rust model integrating a SimAM module in the YOLOv8s backbone and a BiFPN for scale fusion, along with a DWConv for streamlined detection, was developed. The model achieved an accuracy of 94.6%, average accuracy of 91.6%, recall rate of 85.4%, and F1 value of 0.823, outperforming Faster-RCNN and SSD models by 16.35% and 12.49% in classification accuracy, respectively, and detecting a single rust image at 16.18 frames per second. Deployed on mobile phones, the model enables real-time data collection and analysis, supporting effective detection and management of large-scale outbreaks of rust in the field.

## 1 Introduction

Maize is one of the most widely cultivated cereal crops in China, and it is often used as a raw material for animal husbandry, light industry, and health care (Guan et al., 2024). Rust, as a common disease affecting maize plants, propagates under warm and humid conditions, particularly prevalent in both northern and southern cultivation regions of China (Brewbaker et al., 2011). *Puccinia sorghi Schw.* and *P. polysora Underw.* are the causal agents of common corn rust and southern corn rust of maize, respectively. These two diseases represent typical pathogens in the maize production process, exerting significant impacts on crop yield and quality. When rust first appears on a maize plant, it can be identified by the presence of yellow or brown summer spores that form round or oval shapes on the leaves. These spores could be easily spread by the wind and can reproduce quickly, thereby forming a mixture of two types of rust diseases on healthy plants. In the case of a widespread outbreak, common rust typically causes a yield reduction of 20% to 30%, while southern rust can result in losses of up to 50% or even total crop failure (Mei et al., 2023). Although both types of rust may show similar symptoms during the infection stage, their prevalence, distribution, and potential damage levels can vary significantly, and the treatment methods for these two types of rust diseases are different. Therefore, identifying the type of rust accurately that affects maize plants is essential to protect yields, prevent co-infections, realize the use of intelligent detection systems to warn of diseases and thus reduce pesticide use.

Traditional methods for detecting maize rust rely on growers’ experience, reference to plant disease charts, or consultation with plant disease specialists for field observations and identification. However, this approach depends on a large number of plant protection specialists heavily. Variations in experience and knowledge among specialists often hinder disease identification accuracy, resulting in irreparable losses for growers (Lv et al., 2020; Cai et al., 2023). Moreover, molecular identification techniques have been utilized for corn rust detection. Real-time fluorescence quantitative PCR has been proven being effective in distinguishing between *P. polysora* and *P. sorghi* (Crouch and Szabo, 2011).

Although the aforementioned methods have demonstrated a high degree of reliability and accuracy, the cumbersome sample pretreatment, high experimental costs, and long detection cycles have limited their immediate application in field environments. In recent years, optical imaging techniques, with their unique advantages, have gradually become effective tools for early detection and classification of crop diseases. These include infrared imaging (Sinha et al., 2019), hyperspectral imaging (Garhwal et al., 2020), multispectral imaging (Bebronne et al., 2020), and fluorescence imaging (Yang et al., 2024). Due to their non-destructive nature, high speed, and efficiency, these techniques have been widely used in crop disease monitoring. However, despite their ability to provide rich image information, high equipment costs and operational complexity have hindered the widespread adoption of these technologies in routine agricultural practices (Yang et al., 2022). Consequently, it is crucial to develop an economical and efficient diagnostic tool for timely and accurate identification of crop diseases.

Integrated techniques of image processing and deep learning has been applied in maize disease detection studies (Ferentinos, 2018; Khan et al., 2023). The support vector machine demonstrated 83.7% accuracy in classifying common rust, northern leaf blight, and healthy leaves (Aravind et al., 2018). Yu et al. (2021) employed K-means and an enhanced deep learning model, achieving an average accuracy of 93% in diagnosing grey spot, leaf spot, and rust, outperforming VGG and Inception v3. Ahila Priyadharshini et al. (2019) proposed a LeNet network, improving a deep CNN model with 97.89% accuracy in identifying maize images with northern leaf blight, common rust, and grey leaf spot in the PlantVillage dataset. A DenseNet model, based on an optimized CNN architecture, was proposed to identify northern leaf blight, common rust, gray leaf spot, and healthy leaves with 98.06% accuracy (Waheed et al., 2020). Chen et al. (2020) leveraged transfer learning with the INC-VGGN architecture, obtaining a validated accuracy of 91.83% for rice and maize disease recognition. Peng et al. (2021) proposed an improved detection method, which combines the advantages of CNN-based deep feature extraction and support vector machine fusion classification, aiming to quickly and accurately identify three common grape leaf diseases and healthy leaves. An LDSNet model for recognizing maize common rust and big blotch disease in complex background images was designed to reduce computational parameters while effectively improving recognition accuracy (Zeng et al., 2022). A lightweight SSV2-YOLO model based on YOLOv5s was developed to achieve efficient detection of small and high-density sugarcane aphids in unstructured natural environments (Xu et al., 2023). Liu et al. (2024) proposed the MAE-YOLOv8 model to accurately detect small-sized crisp plums in real complex orchard environments. The model accuracy and detection speed reached 92.3% and 68 frames/s, respectively. In order to adapt to the edge computing equipment, the improved lightweight YOLOv8n model was introduced to achieve high-precision real-time peanut leaf disease classification detection (Lin et al., 2024). Sun et al. (2024) introduced CASF-MNet, a novel system that ingeniously combines cross-spatial dimensional feature fusion to enhance accuracy by synergistically fusing color and texture characteristics. Despite these advances, current maize rust detection research faces two challenges, (1) specialized datasets for common rust and southern rust were lacked, and (2) the existing CNN-based detection methods are complex for operation, high demand for computation, and with low accuracy in detection results.

To address the above issues, the main research objectives of this study are, (1) to construct a dedicated dataset for maize common rust and southern rust, and (2) to develop an efficient and accurate target detection algorithm with improved lightweight network structure. The improved model performance on the constructed dataset will be compared with existing methods using multiple evaluation metrics to demonstrate its advantages.

## 2 Materials and methods

### 2.1 Image data acquisition

**Figure 1:**
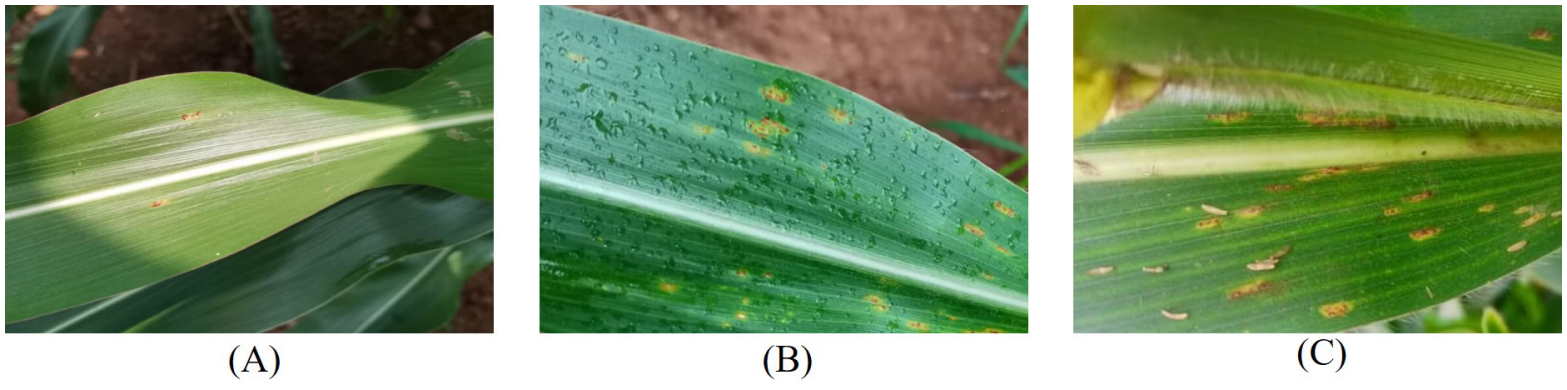
Example of rust image samples in the natural environment. **(A)** Early term rust pathogen infested leaf under sunny day; **(B)** Middle term rust pathogen infested leaf under rainy day; **(C)** Late term rust pathogen infested leaf under cloudy day.

In this study, Zhengdan 958, the most widely planted maize variety in China, was sown and planted at the greenhouse demonstration base in Beibei District, Chongqing, China from September 2022 to January 2023. When the maize plants grew to the 4-6 leaf stage, two types of the rust pathogens, *P. sorghi* Schw. (The causal agent of CCR) and *P. polysora* Underw. (The causal agent of SCR), were inoculated in the middle or tip of the leaves. To ensure that the pathogen can reproduce in large quantities, the ambient temperature was controlled at 22°C -28°C, and the relative humidity was more than 85%. From the second day after pathogen infection, the Canon M50 camera (Resolution: 6000 × 4000 pixels; Producer: Japan) collected leaf images of maize common rust and southern rust from mild to severe infection, between 9:00-11:00 and 15:00-17:00 every day (including sunny, rainy and cloudy weather conditions). In order to enrich the diversity of the dataset, different photographing distance, angles and plant growth stages were also considered during the image collection. All the images were saved as.jpg format. **Figure 1** illustrates the images of rust pathogen infested leaves phenotypes under different meteorological conditions.

### 2.2 Image preprocessing and labeling

**Figure 2:**
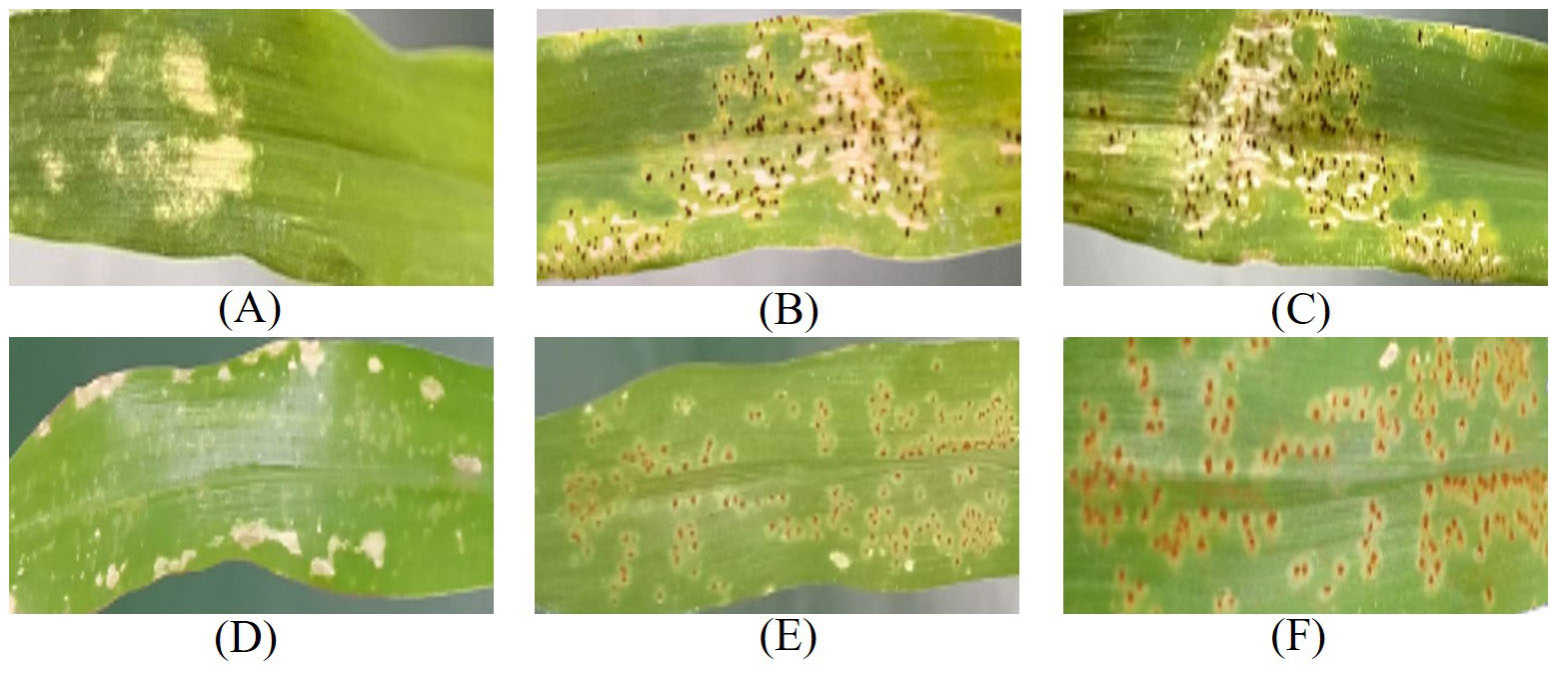
Symptomatic manifestations of the two types of blades. **(A–C)** Represent the performance of CCR after 2, 4, and 6 days of treatment, respectively; **(D–F)** Represent the performance of SCR after 2, 4, and 6 days of treatment, respectively.

The quality of the dataset plays a crucial role in the efficacy of model training. To mitigate manual labeling errors, this study enlisted the expertise of rust disease recognition specialists. The identification of rust disease on plant leaves was based on observable symptoms such as faded green or yellowish spots, along with brown raised spore spots on either the abaxial or adaxial leaf surfaces. For the labeling process, the visual calibration tool LabelImg software was employed to annotate the image dataset. Each infected sample was meticulously labeled after disease type determination, with labels saved in.xml format. **Figure 2** shows the foliar phenotypic symptoms of the two pathogen types after different time treatments.

**Figure 3:**
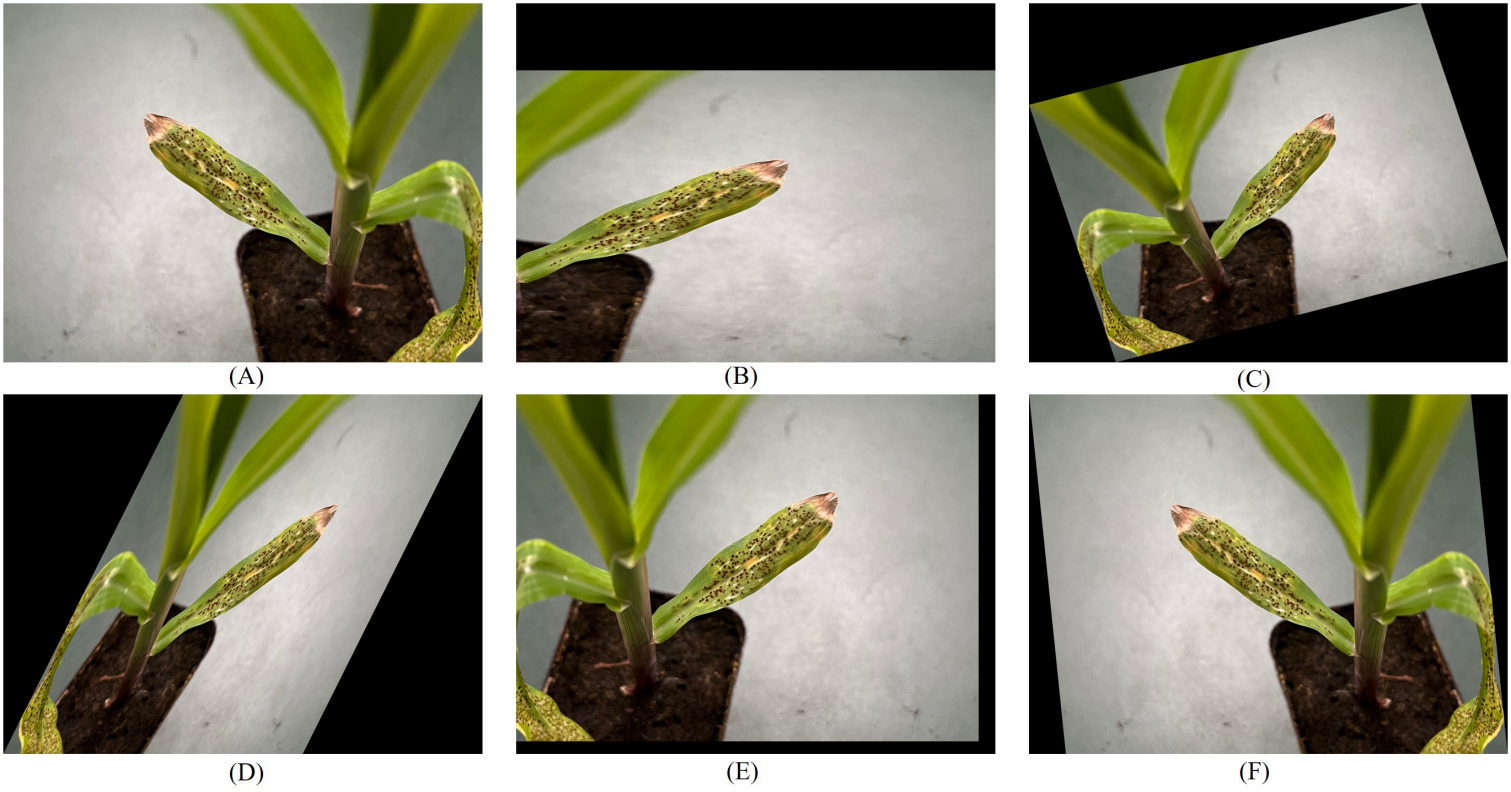
Schematic diagram of image enhancement of rust disease. **(A)** Flip. **(B)** Scale. **(C)** Rotate. **(D)** Shear. **(E)** Translate. **(F)** Flip and Shear.

To thoroughly train the network model, enhance its accuracy, and mitigate overfitting and non-convergence issues, this study implemented various enhancements to the original dataset. By employing image processing techniques, such as flipping, scaling, and rotating (as illustrated in **Figure 3**), the dataset was augmented to 8,730 images. The augmented images were subsequently partitioned into training, validation, and test sets, adhering to a ratio of 7:2:1.

### 2.3 Network model design

YOLOv8 from Ultralytics is an advanced model that extends the capabilities of previous YOLO iterations (Reis et al., 2024), showcasing exceptional accuracy and speed in detection, making it a preferred choice for numerous object detection and image classification tasks. The model architecture comprises an input layer, a backbone network, and a detection head, where the backbone network includes the PANet network and the Detect structure, aimed at achieving feature fusion and target detection specifically for rust detection. In the PANet framework, fundamental components such as FPN and PAN play critical roles. FPN extracts feature layer information from top to bottom, facilitating the fusion of details from higher and lower layers to enhance the network’s ability to detect objects of varied sizes. Conversely, PAN operates by extracting features from bottom to top, ensuring precise positional information retrieval. Additionally, the Detect structure, featuring three branches of differing sizes, generates classification results and target coordinate data.

**Figure 4:**
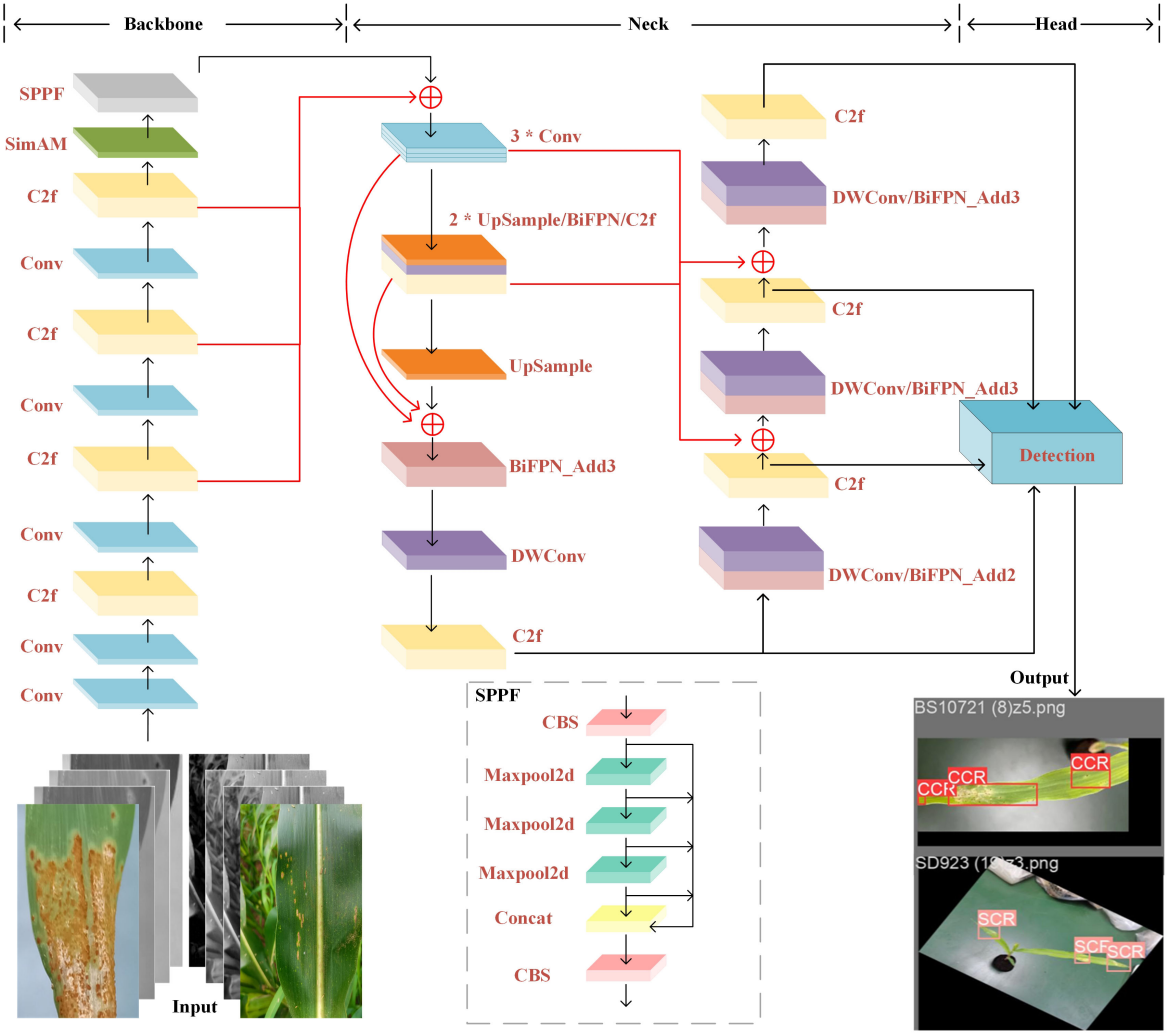
The Maize-Rust model– identifying common rust and southern rust in maize specifically.

In this study, a Maize-Rust model based on YOLOv8 network was designed for the identification of common rust and southern rust. As shown in **Figure 4**, the SimAM, BiFPN, and DWConv structures were integrated into the YOLOv8 backbone and necking network effectively. This enhanced network not only boosted detection capability and speed but also mitigated false alarms and missed detections within the model.

#### 2.3.1 SimAM architecture

When using the YOLOv8 model to extract the characteristics of CCR and SCR, the model often fails to achieve the expected detection results due to the subtle phenotypic changes and feature leakage of these diseases. In order to overcome this challenge, this study introduces the SimAM attention mechanism (Park et al., 2020), so that the network can focus on detailed feature information more quickly and integrate it into the backbone network of YOLOv8. It helps the network dynamically adjust the degree of attention to different features by calculating the saliency of each feature point. In the process of detecting corn rust, SimAM can guide the network to capture rust pixels more keenly, thereby improving the accuracy of feature extraction. In addition, SimAM also enhances the recognition ability of the backbone network to disease features, enabling the network to focus more on the key features related to the disease, such as the shape, color and texture of the lesion. This fine capture and attention to disease features not only improves the accuracy of target detection, but also enhances the ability of the model to identify similar features in the image, and ultimately improves the performance of the model in distinguishing CCR and SCR.

**Figure 5:**
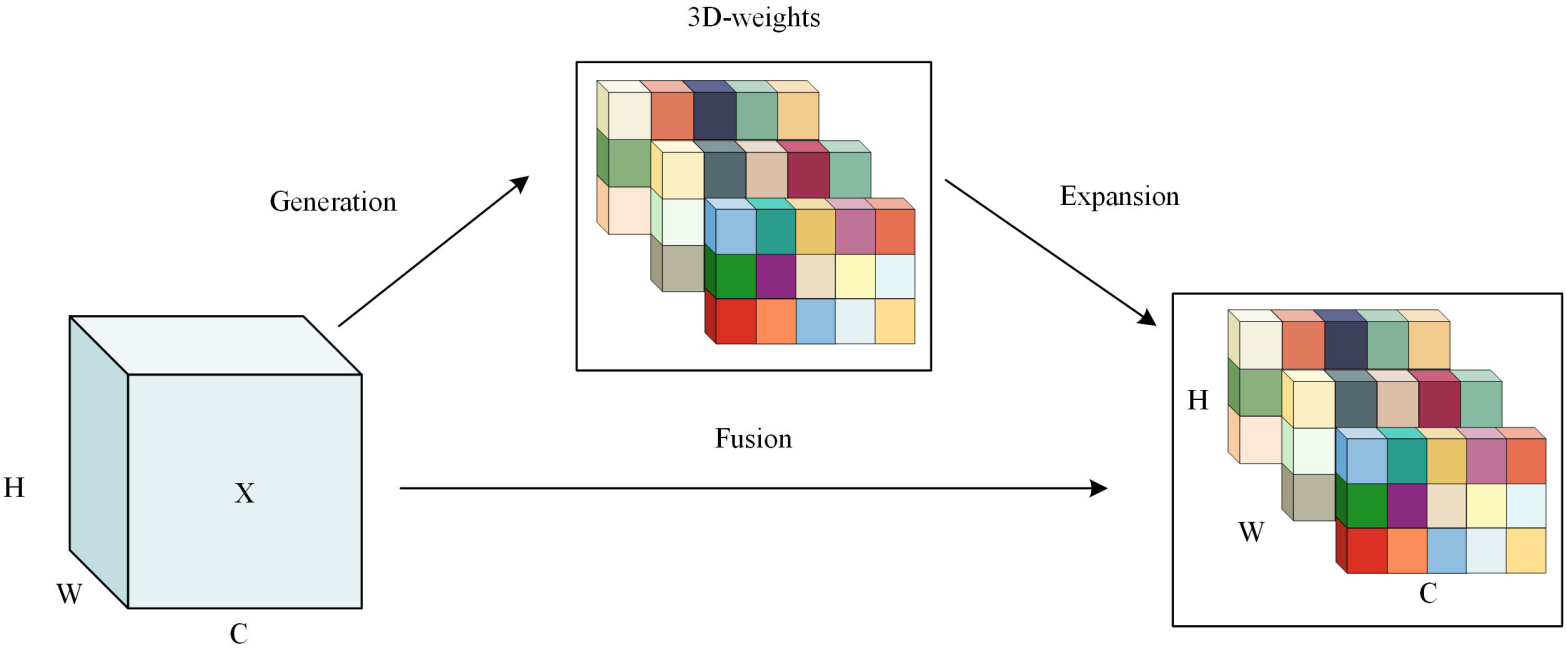
The Similarity-Aware Attention Mechanism structure.


The SimAM mechanism is designed to heighten the model’s sensitivity to patterns of similarity within the input image. Distinct from traditional attention modules like CBAM (Woo et al., 2018), SE (Hu et al., 2019), and CA (Hou et al., 2021), the SimAM module uniquely captures both spatial location information and pixel-level similarity features, effectively guiding the network to concentrate on critical regions in the image. As depicted in **Figure 5**, its structure employs an energy function to generate 3D weighted attention, facilitating linear separability between the target neuron and other neurons. The minimum energy function can be defined as follows:

(1) $${\text{e}}_{\text{t}}=\frac{4\left({\text{δ}}^{2}\text{+λ}\right)}{{\left(\text{t-μ}\right)}^{2}{+2\delta }^{2}+2\lambda }$$

where *t* denotes the target neuron in the single-channel input feature; *μ* and *δ^2^* symbolize the average and variability of all neurons; and *λ* represents the weight constant.

The smaller *e_t_* means that the target neuron *t* is more different from the surrounding neurons, indicating a higher importance in the image processing process. In addition, to refine the feature process, a scaling operator was used the module instead of an additive operation. The refinement stage could be expressed as the following Equation 2:


(2) $$\tilde{X}=sigmoid\left(\frac{1}{E}\right)⨀X$$

where *E* denotes the grouping of all *e_ts_* in the channel and spatial dimensions; the overvalues in *E* are limited by the inclusion of a Sigmoid function, which do not affect the relative importance of each neuron because it is a monotonic function.

#### 2.3.2 Optimizing the neck network

##### 2.3.2.1 BiFPN feature fusion network

**Figure 6:**
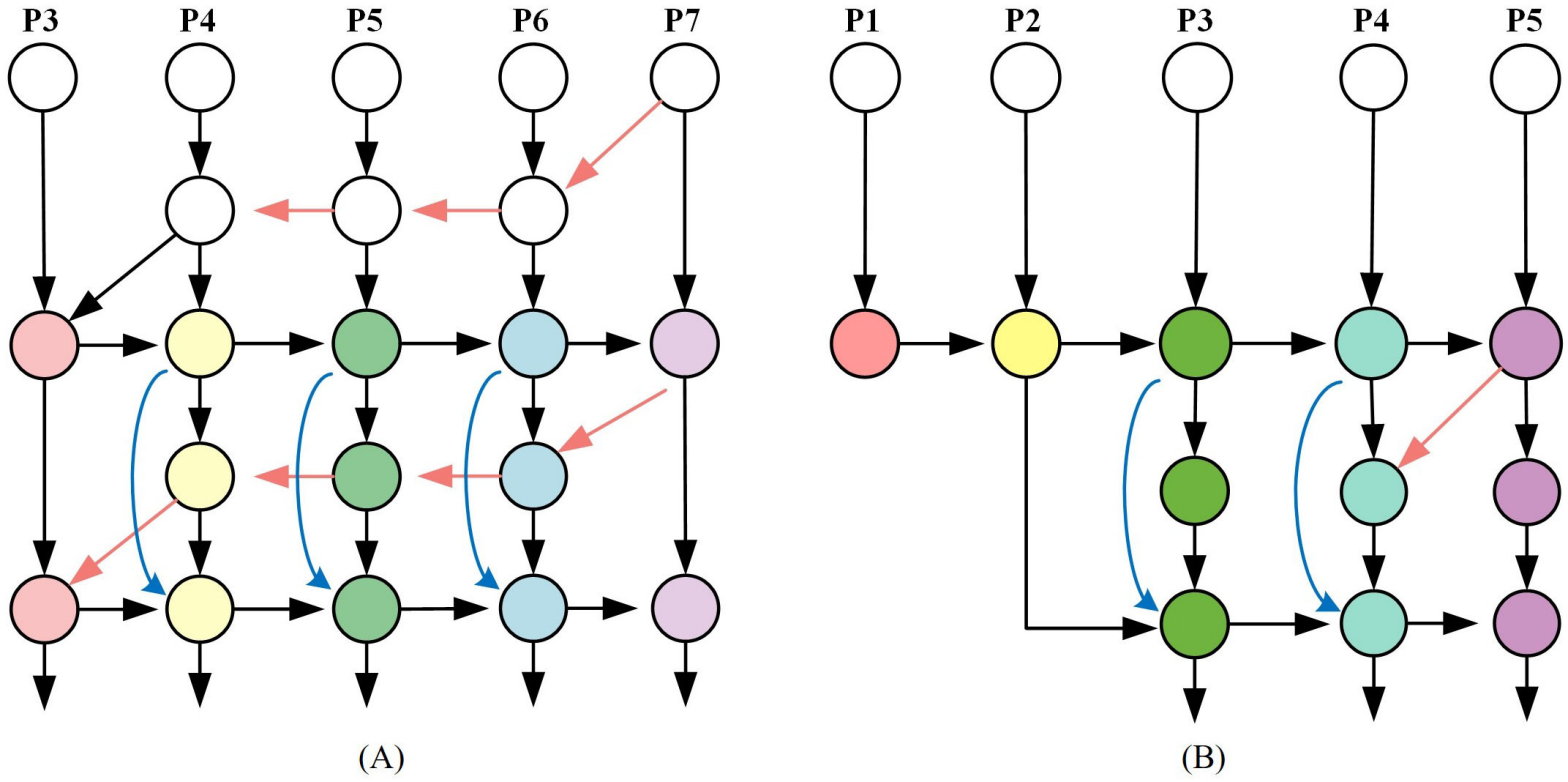
BiFPN structure. **(A)** original BiFPN structure. **(B)** added P2 to BiFPN structure. Note: P1 to P7 represent the input feature maps corresponding to different levels.

YOLOv8 utilizes multi-resolution feature images to detect objects of different sizes. Deeper layers provide rich semantic information and wide receptive fields, making them suitable for detecting larger objects. However, due to the small size of corn leaves and disease spores, along with YOLOv8’s significant down sampling, extracting detailed information from deeper features is challenging, leading to missed detections or inaccuracies for small targets. To improve model efficiency, this study integrated the weighted BiFPN (Tan et al., 2020), enhancing the fusion of features from different resolutions. The modified BiFPN architecture, shown in **Figure 6A**, retained feature fusion from two to five layers. In addition, by extending the resolution of the feature map to 160 × 160, BiFPN further enhances the detection ability of the model for small targets. The larger feature map provides more spatial details, which enables the model to locate small targets more accurately and reduce the missed detection rate. At the same time, cross-scale connection on the initial three layers not only enhances the ability of small target detection, but also reduces the loss of early rust detection, because rust and other diseases often appear as small spots or discoloration in the early stage, and these features are more easily captured on higher-resolution feature maps. As shown in **Figure 6B**.

During feature fusion, variations in input feature resolution led to unequal contributions to outputs. When fusing size features, the Resize method was typically employed to equalize feature sizes before summation. Taking layer 6 of the feature map as an example, its intermediate feature $P_{6}^{td}$ and output feature $P_{6}^{out}$ are specifically calculated as:


(3) $$P_{6}^{td}=Conv\left(\frac{\omega_{1}\cdot P_{6}^{in}+\omega_{2}\cdot Resize\left(P_{7}^{in}\right)}{\omega_{1}+\omega_{2}+\epsilon }\right)$$

(4) $$P_{6}^{out}=Conv\left(\frac{\omega_{1}^{'}\cdot P_{6}^{in}+\omega_{2}^{'}\cdot P_{6}^{td}+\omega_{3}^{'}\cdot Resize\left(P_{5}^{out}\right)}{\omega_{1}^{'}+\omega_{2}^{'}+\omega_{3}^{'}}\right)$$

where $P_{i}^{in}$ lies the input feature at the ith level.

Given the varying importance of features of different sizes in the output, enhancing feature fusion is crucial. This is achieved by introducing a learnable weight parameter and normalization to balance the weights. Integrating the BiFPN framework and the P2 layer for small target detection improves the model’s performance on this dataset, albeit with increased computational complexity.

##### 2.3.2.2 Introduce the DWConv module

In the neck network’s feature fusion process, various parameters affect fusion speed. This study utilized the Depthwise Separable Convolution (Kaiser et al., 2017) structure as an alternative to traditional Conv modules. Compared with traditional convolution, DWConv significantly reduces the computational complexity and the number of parameters by separating channels and spatial dimensions. Specifically, the traditional convolution needs to consider both channel and spatial information, so its calculation and parameter quantity are relatively high. DWConv achieves effective decomposition and reduction of computational complexity by separating these two dimensions. Since DWConv applies filters to each channel independently in the deep convolution stage, it can also use the sparsity of the input feature map to further reduce the amount of computation.

**Figure 7:**
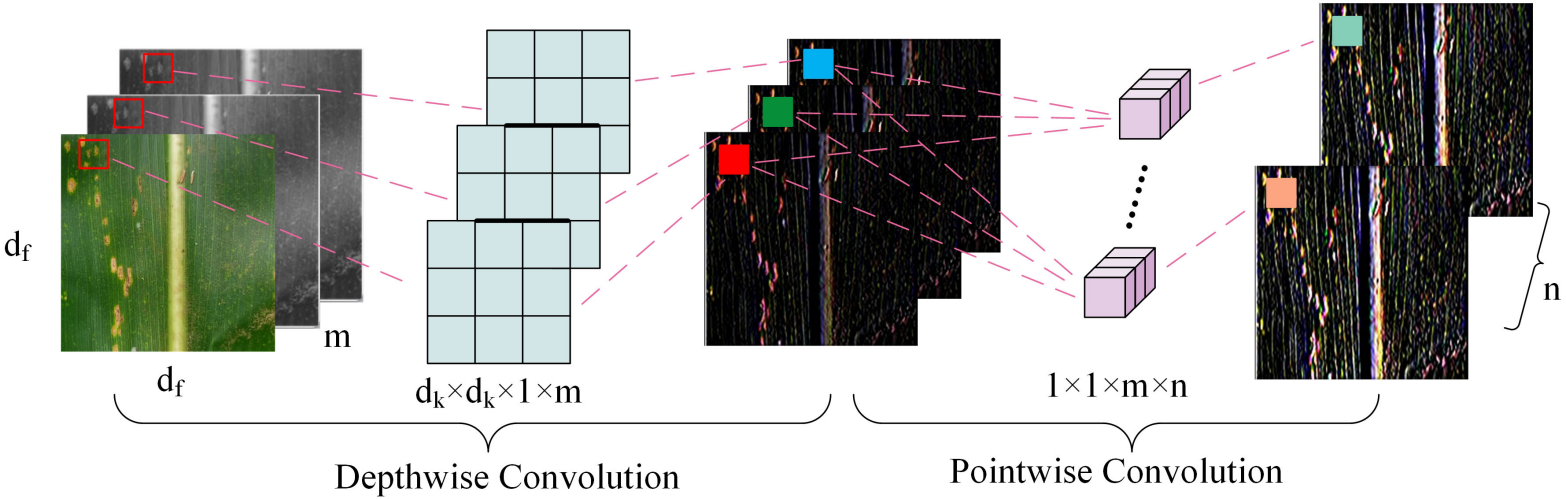
The Lightweight DWConv Structure.

**Figure 7** illustrates how DWConv divided standard convolution into two phases: Depthwise convolution applies a lightweight single-channel filter to each input channel, followed by Pointwise convolution or 1×1 convolution to combine input channels for new features. Unlike conventional convolution, DWConv separates channel and spatial dimensions, enhancing optimization by handling these correlations independently. Moreover, depth-separable convolution incorporates grouped convolution, significantly reducing parameter count.

For a standard convolutional layer, assuming that *m* is the number of channels for the input layer, *n* is the number of channels for the output layer, *d_f_* is the input feature map size, and *d_k_* is the convolutional kernel size. Thus, the total computation for this convolutional layer could be calculated as:


(5) $$C=d_{f}\times d_{f}\times m\times n\times d_{k}\times d_{k}$$

During deep convolution, since each convolution kernel processes each channel individually, there will be m convolution kernels to process the image, with a parameter computation CD for each kernel defined as:


(6) $$C_{D}=d_{k}\times d_{k}\times 1\times m\times d_{f}\times d_{f}$$

Then, the generated feature maps are subjected to a 1 × 1 conventional convolution process. The parameter computation CP for point-by-point convolution is:


(7) $$C_{p}=1\times 1\times m\times n\times d_{f}\times d_{f}$$

The ratio to the computational effort of a standard convolutional layer is:


(8) $$C^{\prime }=\frac{C_{DWConv}}{C}=\frac{C_{D}+C_{P}}{C}=\frac{1}{n}+\frac{1}{d_{k}^{2}}$$

According to Equation 8, when the ratio $C^{\prime }$ is less than 1, it indicates that the introduction of DWConv leads to a reduction in the number of overall model parameters. The reduction in model parameters leads to a lighter and more efficient network architecture.

### 2.4 Training environment and evaluation index

#### 2.4.1 Hardware and software parameters

This study utilized a Tencent Cloud GPU computing-based server (located in the Chengdu data center) for model training, with specific parameters as follows: Ubuntu 20.04 operating system, two NVIDIA Tesla T4 GPUs, an Intel Xeon Cascade Lake CPU with a frequency of 2.5 GHz, and 160 GB of RAM. The software environment included CUDA 11.0, Python 3.9, and Pytorch 2.0.

The network model was optimized using the SGD algorithm for training the maize rust disease recognition model. SGD estimates the gradient using a small number of samples per iteration, reducing computational cost and speeding up training, making it ideal for large datasets. The learning rate was set to 0.001, with 150 epochs and input images of 640 × 640 pixels per batch to meet the model’s requirements.

#### 2.4.2 Evaluation indicators

To evaluate the model’s detection performance, precision (P), recall (R), mean average precision (mAP), F1-score (F1), single-image detection time (dR), computational complexity (FLOPs), Parameters, and model weights (Size) are used as experimental evaluation metrics in this paper. The specific calculation formula is as follows:


(9) $$P=\frac{M_{TP}}{M_{TP}+M_{FP}}\times 100\%$$

(10) $$R=\frac{M_{TP}}{M_{TP}+M_{FN}}\times 100\%$$

(11) $$mAP=\frac{\sum^{\;}AP}{N}=\frac{\sum^{\;}\int_{0}^{1}P\left(R\right)dR}{N}$$

(12) $$F1=2\times \frac{P_{M}\cdot R_{M}}{P_{M}+R_{M}}$$

where *M_TP_* denotes the number of rusts correctly detected as rusts, *M_FP_* denotes the number of rusts incorrectly detected as rusts, and *M_FN_* denotes the number of rusts missed in the image. *AP* is the mean function of P for all R-values between 0 and 1, and *N* is the number of detection categories. In this study, the recognition targets were only two categories, so *N* is 2.

## 3 Results

### 3.1 Comparison of benchmark model performance

In order to detect the two types of rust diseases accurately and efficiently, this study adopts the YOLOv8 model as the benchmark and trains the five versions of the model sequentially, with the specific results detailed in **Table 1**.

**Table 1:** Comparison of the performance of the original YOLOv8 model.

Methods	Size (MB)	Precision (%)	Recall (%)	mAP
YOLOv8n	6.2	84.3	64.7	0.730
YOLOv8s	22.5	87.5	71.0	0.771
YOLOv8m	52.0	92.7	78.6	0.860
YOLOv8l	87.6	95.1	79.0	0.866
YOLOv8x	134.76	94.2	79.7	0.867

**Table 1** data showed that the mAP of the YOLOv8s model surpassed YOLOv8n by 5.62% but fell below YOLOv8m, YOLOv8l, and YOLOv8x by 10.35%, 10.97%, and 11.07%, respectively. Additionally, the YOLOv8s model’s weight size was smaller than YOLOv8m, YOLOv8l, and YOLOv8x by 29.5MB, 65.1MB, and 112.26MB, respectively, yet only 16.3MB larger than YOLOv8n. Therefore, to balance detection accuracy and network efficiency, the YOLOv8s model was chosen as the benchmark for future research.

### 3.2 Performance comparison of experiments introducing attention mechanisms

This study utilizes the YOLOv8s model, incorporating five attention mechanisms - CA, CBAM, CPCA, SE, and SimAM - into the backbone network. The detection model is trained until convergence, with no alterations to the remaining network or parameters. The performance results are detailed in **Table 2**.

**Table 2:** Results of the effect of attention mechanisms on model performance.

Methods	Precision (%)	F1	mAP	FLPOs (G)
YOLOv8s	87.5	0.784	0.771	28.6
YOLOv8s + CA	83.8	0.754	0.759	50.1
YOLOv8s + CBAM	73.6	0.669	0.678	29.3
YOLOv8s + CPCA	88.5	0.779	0.774	29.1
YOLOv8s + SE	86.2	0.757	0.750	28.2
YOLOv8s **+ SimAM**	**90.0**	**0.794**	**0.795**	**28.6**

**Table 2** shows that the model YOLOv8s + SimAM represented by this value shows the best results after multiple experiments.

**Table 2** illustrated that incorporating the SimAM attention mechanism in the YOLOv8s network architecture outperformed the other four attention mechanisms and the original model in all aspects. Specifically, the SimAM model achieved a 1.5% increase in detection accuracy compared to the original model, and a 1% enhancement in the F1 index. Compared to the other four attention mechanisms, the SimAM model showed detection accuracy improvements ranging from 1.5% to 16.4% and mAP50 metric enhancements from 2.1% to 11.7%.

### 3.3 Ablation test of the model

To investigate the effects of the SimAM attention mechanism, BiFPN small target detection, and DWConv module on rust detection performance, three structures were introduced into the official YOLOv8s network. The results of the ablation tests were presented in **Table 3**, with evaluations focused on precision, average precision, F1 score, floating-point operations, and number of parameters to assess model efficiency.

**Table 3:** Ablation tests using different modules.

No.	SimAM	DWConv	BiFPN	P (%)	mAP50	F1	FLOPs (G)	Params (M)
1				91.1	0.771	0.818	28.6	11.14
2	√			90.0	0.795	0.794	28.6	11.13
3		**√**		90.6	0.812	0.822	7.3	2.62
4			**√**	91.2	0.805	0.816	48.7	21.02
5	**√**	**√**		90.5	0.810	0.814	16.9	10.86
6	**√**		**√**	91.3	0.814	0.822	8.6	29.75
7		**√**	**√**	91.2	0.813	0.790	25.0	7.37
8	√	√	√	**94.6**	**0.916**	**0.823**	**20.6**	**4.11**

Bold fonts represent the best group of multiple evaluation indicators in 8 groups of experiments.

**Table 3** displayed the impact of different trials on the model. In Trial 2, integrating the SimAM attention mechanism led to a 3.11% increase in mAP50 with minimal parameter reduction. Trial 3 introduced DWConv, reducing parameters and floating-point operations by 75% due to the combined DW and PW structure, resulting in a 0.49 percentage point increase in F1 scores and a 5.31% average accuracy boost. Trial 4 incorporated BiFPN, enhancing average accuracy by 4.40% and increasing parameters by 47.03%. The combined use of improved modules in Tests 5, 6, and 7 resulted in changes in mAP of -0.65%, 2.19%, and 1.09%, respectively. Ablation tests confirmed the positive impact of the enhancements on YOLOv8s target detection, improving the model accuracy in identifying rust diseases in maize while maintaining its lightweight design.

### 3.4 Model feature visualization

**Figure 8:**
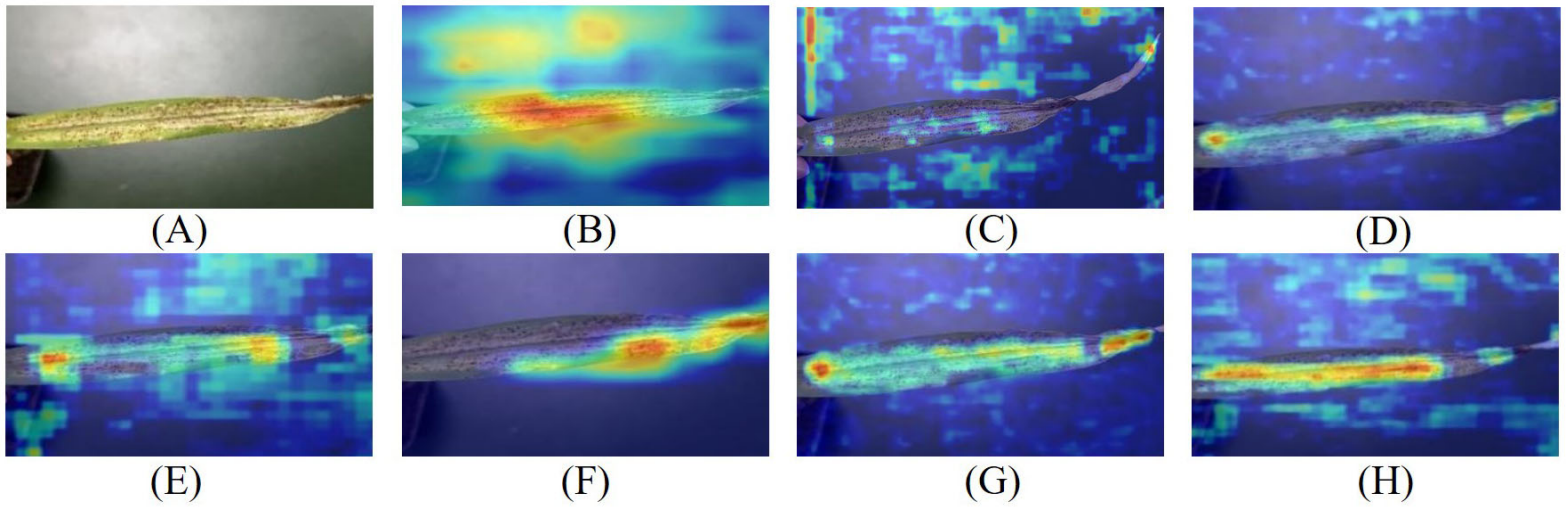
Common rust image visualization features. **(A)** Original image. **(B)** YOLOv5. **(C)** YOLOv7. **(D)** YOLOv8. **(E)** YOLOv8+SimAM. **(F)** YOLOv8+DWConv. **(G)** YOLOv8+BiFPN. **(H)** Maize-Rust.

**Figure 9:**
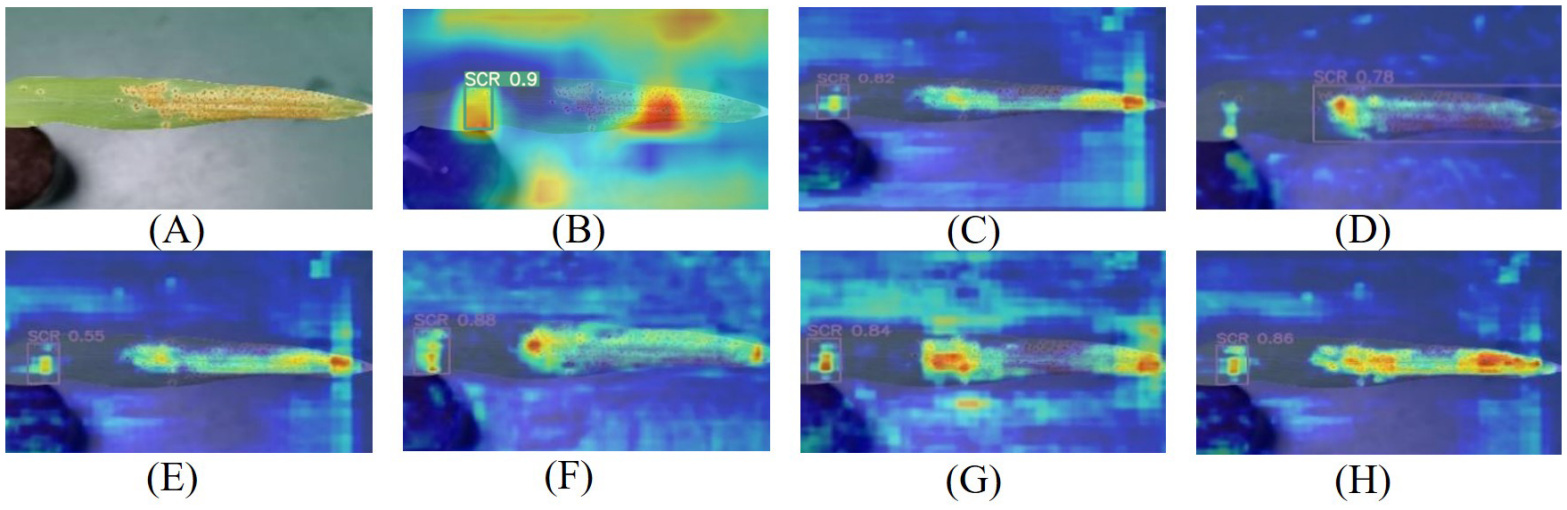
Southern rust image visualization features. **(A)** Original image. **(B)** YOLOv5. **(C)** YOLOv7. **(D)** YOLOv8. **(E)** YOLOv8+SimAM. **(F)** YOLOv8+DWConv. **(G)** YOLOv8+BiFPN. **(H)** Maize-Rust.

To observe the Maize-Rust model’s recognition ability more intuitively, Grad-CAM was utilized to generate the heat maps. Upon comparing the heat map results of the seven detection models in **Figures 8**, **9**, it was clear that the Maize-Rust model showed higher color intensity in the leaf rust spot region compared to the other six models significantly. This suggested that the network prioritizes areas with rust during detection, even highlighting small rust spots. The incorporation of the SimAM module, BiFPN module, and DWConv structure had improved the model’s accuracy in detecting the target while reducing the impact of incorrect samples on overall prediction. This enhancement directed the model’s focus to the specific characteristics of rust disease, thereby enhancing its ability to detect rust disease.

### 3.5 Comparison test of different network models

To verify the performance of various models in detecting two types of rust diseases in maize visually, many deep learning-based detection algorithms were selected for the tests in this study. The experimental results of Faster-RCNN, SSD, EfficientDet, RetinaNet, YOLOv3, YOLOv5, YOLOv7, YOLOv8, YOLOv9, YOLOv10 and YOLOv11 were presented in **Table 4**.

**Table 4:** Comparing the results of different algorithm models for rust image recognition.

Model	d_R_ (ms)	P (%)	R (%)	F1	mAP	Size (MB)
Faster-RCNN	410.4	81.3	62.8	0.709	0.689	108.0
SSD	582.6	84.1	65.1	0.734	0.813	42.4
EfficientDet	81.3	87.1	67.6	0.761	0.749	21.4
RetinaNet	91.5	87.7	68.4	0.768	0.757	50.6
YOLOv3	314.8	88.6	70.2	0.783	0.785	123.5
YOLOv5	160.4	83.0	64.2	0.724	0.713	14.4
YOLOv7	310.2	62.1	61.7	0.619	0.678	71.3
YOLOv8	282.0	91.1	74.2	0.818	0.771	22.5
YOLOv9	75.4	78.9	55.4	0.645	0.638	19.4
YOLOv10	94.4	85.2	65.6	0.741	0.732	20.5
YOLOv11	72.4	90.0	71.9	0.799	0.801	19.2
**Maize-Rust**	**52.8**	**94.6**	**85.4**	**0.823**	**0.916**	**19.7**

The bold style in **Table 4** shows that the model proposed in this paper is the best compared with multiple detection models.

**Table 4** showed that Maize-Rust improves precision by 16.35%, recall by 16.07%, and mAP by 32.94%, with a notable reduction in detection time per rust image compared to SSD. The Maize-Rust model outperformed Faster-RCNN across all evaluation metrics. Compared with EfficientDet and RetinaNet, the single image detection time of Maize-Rust is reduced by 28.5 and 38.7 ms, respectively, and the weight is relatively close. Notably, in recall metrics, Maize-Rust model demonstrated significant enhancements compared to YOLOv3, YOLOv5, YOLOv7, YOLOv8, YOLOv9, YOLOv10 and YOLOv11, with improvements ranging from 15.09% to 54.1%. Similarly, in mAP metrics, improvements ranging from 15.94% to 43.5% were observed compared to YOLOv3, YOLOv5, YOLOv7, YOLOv8, YOLOv9, YOLOv10 and YOLOv11. The proposed model achieved the highest F1 score among YOLO networks and had weights comparable to YOLOv5, YOLOv9, YOLOv10 and YOLOv11, but lighter than YOLOv3, YOLOv5, and YOLOv7. Regarding detection time per rust image, the suggested model cut down by 357.6ms, 529.8ms, 262ms, 107.6ms, 257.4ms, 229.2ms, 22.6ms, 41.6ms and 19.6ms in comparison to the Faster-RCNN, SSD, YOLOv3, YOLOv5, YOLOv7, YOLOv8, YOLOv9, YOLOv10 and YOLOv11 models. The results highlighted the superior performance of the Maize-Rust model in terms of accuracy and speed for corn rust visual recognition.

**Figure 10:**
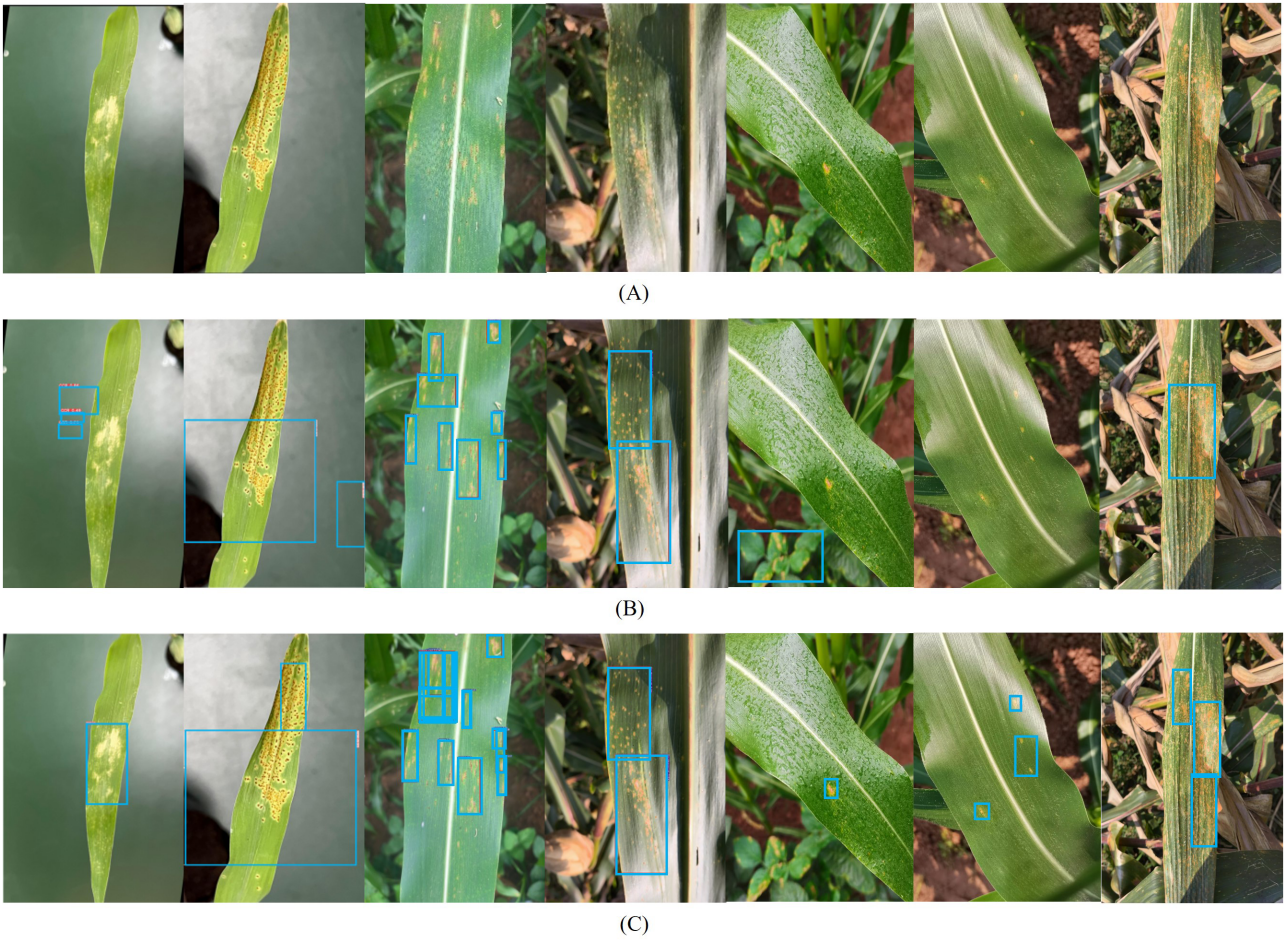
Detection results in different scenarios and climates. **(A)** Images of two species of rust leaves in indoor and field environments. **(B, C)** the results for YOLOv8 and Maize-Rust model identifying rust.

Due to the inconsistent background complexity of corn rust plants in greenhouse and field environments and the varying growth patterns of corn leaves, the baseline model and Maize-Rust model were employed to identify common rust and southern rust leaves in these environments, with specific detection results illustrated in **Figure 10**.

**Figure 10** substantiated the efficacy of the proposed approach alongside other detection methodologies in both real-world and greenhouse settings. The results revealed that, despite the similar background to leaf color in greenhouses, the proposed method outperformed other model in the validation dataset. Furthermore, the missed detection rate of the model under the three climatic conditions in the field was still lower than that of other detection methods. This demonstrated the robustness of the developed Maize-Rust model in reliably detecting common and southern rust in both field conditions and the dataset. Therefore, through the comprehensive comparison of all indicators, Maize-Rust is more suitable for the detection of maize rust in different environments.

In the identification of early rust, the model demonstrated a relatively low performance. Upon verifying and analyzing the early disease map, it became apparent that the model could easily misjudge early rust as healthy leaves or fail to detect it. This issue may stem from several factors. Firstly, the characteristics of early rust are not distinct, making it susceptible to confusion with the background of healthy leaves. Secondly, the features of early rust are prone to interference from the surrounding environment, particularly in complex field settings. Lastly, variations in illumination conditions significantly impact image quality, thereby affecting the accuracy of feature extraction. To address this challenge, future research will focus on implementing model integration techniques and optimizing feature extraction to enhance the model’s capability in identifying early rust features. Additionally, the exploration of data augmentation strategies to simulate lesions under diverse lighting conditions aims to enhance the model’s performance in low-light situations.

### 3.6 Model deployment and practical application

**Figure 11:**
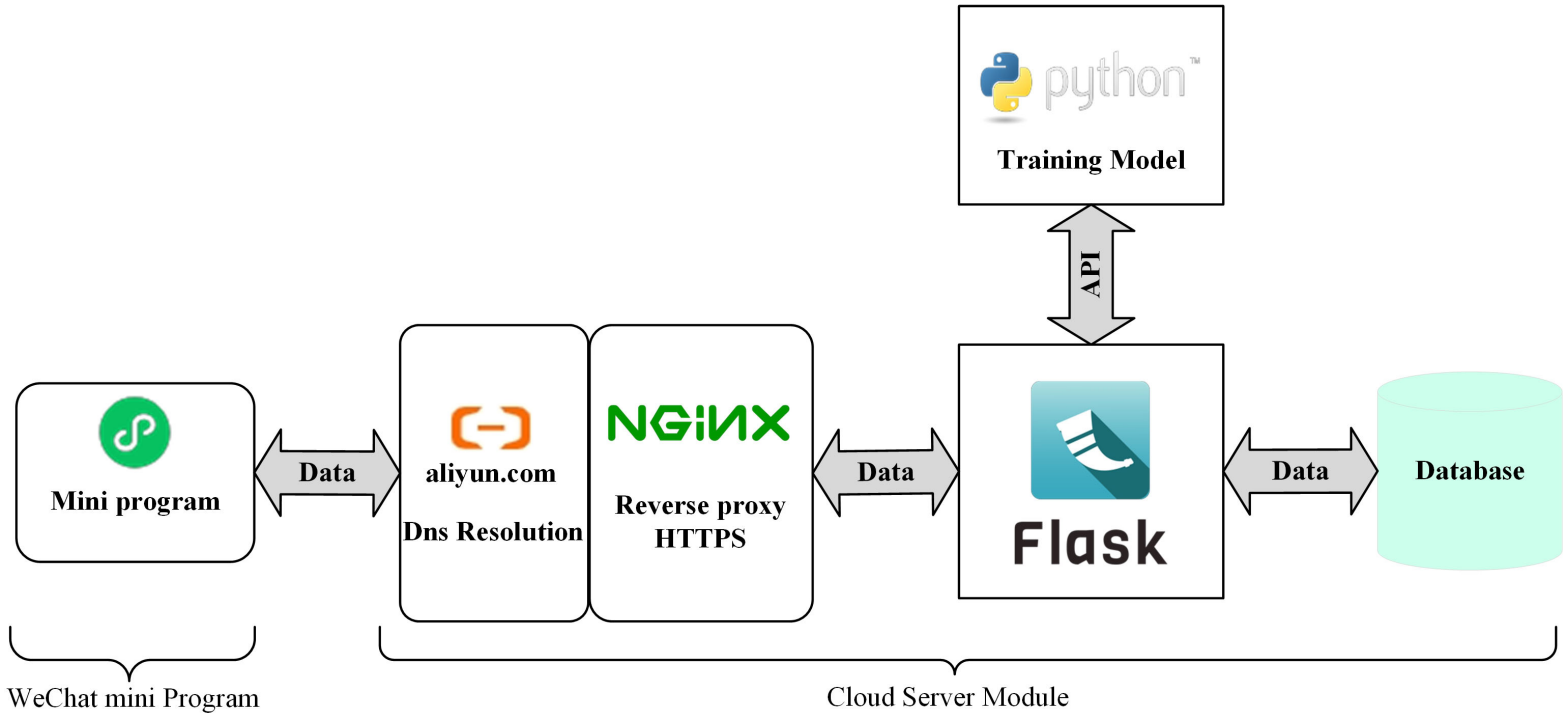
Framework of maize rust identification system.

Although Maize-Rust can accurately and quickly detect two types of corn rust, it has limitations in field environment detection. In order to verify the practical application performance of Maize-Rust, control the use of pesticides and realize early rust monitoring and early warning in the field, this study developed a corn rust recognition system based on the “cloud server and terminal intelligence” architecture. The combination of Ali cloud and WeChat applet can quickly solve the problem of server deployment, optimize server balance and expansion, and provide the necessary service deployment. The architecture of corn rust recognition system includes WeChat applet module and cloud server module. When users use small programs, they need to take or select rust pictures through the WeChat small program, and then upload them to the cloud server module. The running background service can process the uploaded data and then return the data analysis results to the user (**Figure 11**).

**Figure 12:**
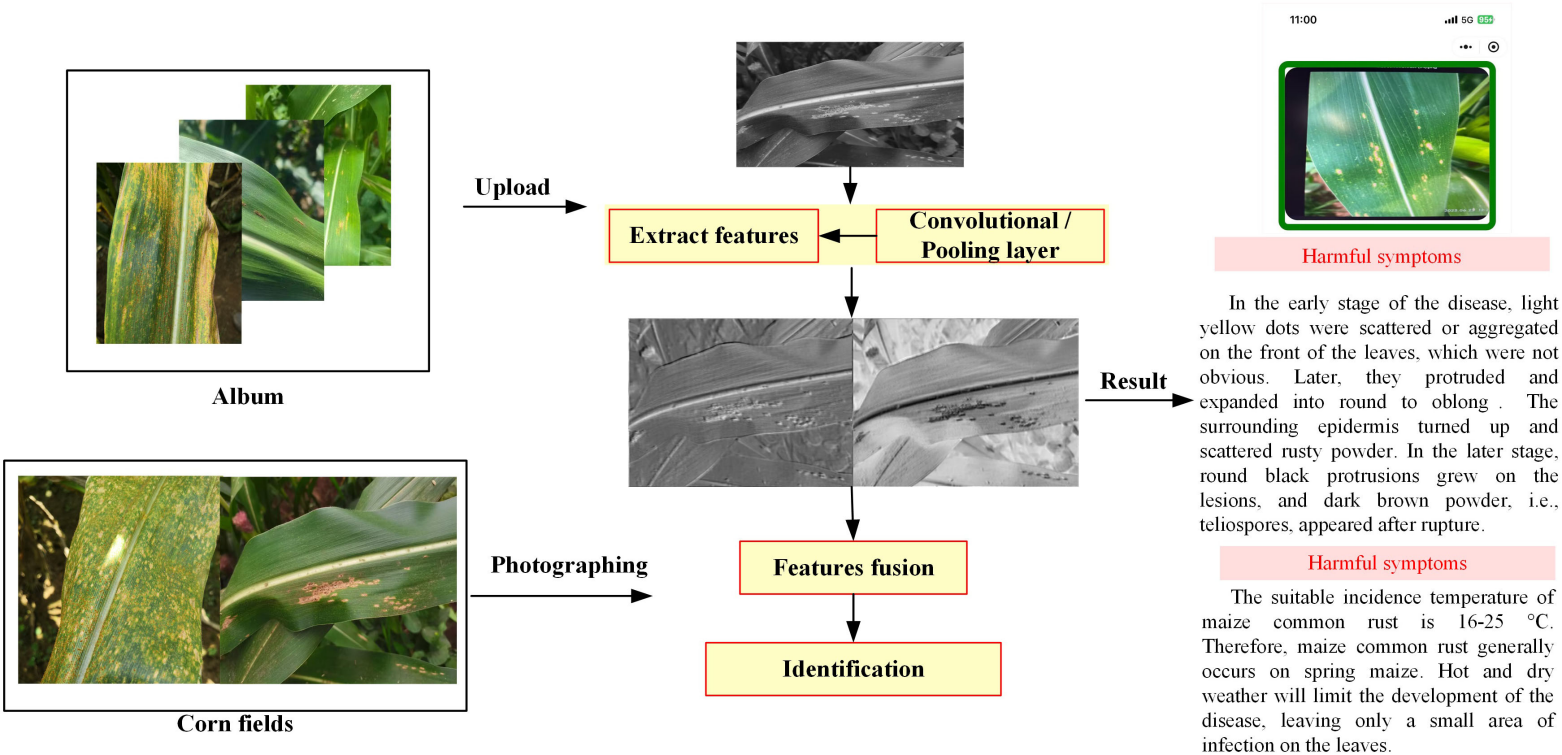
The actual test of Maize-Rust model on mobile devices.

The actual test of Maize-Rust proposed on mobile devices is shown in **Figure 12**. Users can choose to take photos or upload photo album disease images. Then the Maize-Rust weight file is executed and the corresponding label is returned to the user as a result. In addition, the program also outputs rust phenotypic symptoms and control methods to help users identify maize diseases in the field.

## 4 Discussion

In this study, the SimAM, BiFPN, and DWConv modules were incorporated into YOLOv8s algorithm to enhance their performance, successfully enabling the detection of common and southern maize rusts.

In Section 3.1, the YOLOv8 algorithm’s five model classes, increasing in weight size and depth, showed improved precision and recall during training for both YOLOv8l and YOLOv8x versions. This improvement was attributed to the network’s increased depth, expanding parameter capacity to capture more features and patterns, enhancing rust image detection. However, the deeper network also raised computational demands, prolonging inference and detection times and requiring more hardware resources. Balancing detection accuracy and computational load is crucial in practical applications.

The YOLOv8s model served as the baseline for improved models, allowing a comparative analysis of detection performance on the same dataset. Slower detection speeds of popular algorithms like Faster-RCNN (Ren et al., 2017), SSD (Liu et al., 2016), YOLOv3 (Redmon and Farhadi, 2018), YOLOv7 (Wang et al., 2023) and YOLOv9 (Wang et al., 2024) highlighted their limitations in real-time field detection tasks. In contrast, the YOLOv8 model demonstrated superior performance in corn rust detection, consistent with prior studies (Zhao et al., 2022). YOLO superior performance was attributed to its simultaneous classification and regression capabilities, outperforming two-stage algorithms in speed. Furthermore, YOLOv8’s enhanced feature extraction techniques, including anchorless and SimOTA, along with its efficient skeleton extraction network and neck structure, improved feature layer utilization in rust image analysis.

The SimAM module enhanced the convolution layer ability to extract object features and improve model detection reliability. Previous studies had explored the application in object detection tasks (Zhao et al., 2021; Wang et al., 2022; Geng et al., 2024; Meng et al., 2024). These studies utilized attention mechanisms including CA, CBAM and SimAM to enhance convolutional layers focus on target features and extract more feature pixels. The reasons why SimAM module excelled other mechanisms may be as follows: (1) By adapting to various feature layers and weighting feature information, the model could better capture rust disease details and suppress noise. (2) 3D attention weights were calculated using pixel correlations in the feature map to enhance feature recognition with local structural information. Moreover, the module’s analytic solution for weight computation introduced no new parameters, maintaining model complexity.

The BiFPN module improved rust detection in small pixel areas for several reasons: (1) Feature fusion across layers allowed the YOLOv8s model to effectively use semantic information, enhancing its ability to capture detailed rust pixel information. (2) BiFPN incorporated bidirectional information transfer, promoting full communication between target features and enhancing responsiveness to small targets. (3) The module’s adaptive feature adjustment enabled dynamic self-adjustment, improving detection accuracy for small objects. (4) Including the P2 layer in the BiFPN structure facilitated extraction of deeper feature maps, providing richer spatial information and strengthening the network’s ability to detect small targets. These findings are consistent with prior research (Chen et al., 2024; Xie et al., 2023).

The DWConv module controlled model computational parameters effectively, which was similar to the results of previous studies (Liu et al., 2023). This advantage could be attributed to: (1) Depth-separable convolution, compared to traditional convolution, separates spatial and channel correlations, resulting in reduced parameters and enhanced computational efficiency. (2) By exploiting feature independence between channels, the DWConv enables more detailed spatial feature extraction through channel-specific processing, facilitating the accurate differentiation between rust types. The integration of the BiFPN structure in the backbone necessitates a balance with lightweight components to minimize computational footprint.

Consequently, the Maize-Rust model optimally combined performance, computational cost, and speed, outperforming mainstream alternatives. The proposed model significantly improved performance in small object detection and lightweight deployment compared to previous models, which often struggled with these tasks due to their limited capability in handling small objects and optimizing for low-resource environments. By addressing these limitations, our work introduces novel techniques that enhance detection accuracy and reduce computational overhead. However, it’s important to note that, while our model excels in recognizing maize leaf diseases, its dataset scope is limited to two types, leaving room for broader coverage. To mitigate overfitting, we augmented image data, yet the improvement was constrained by the lower image clarity of the augmented samples.

## 5 Conclusion

Addressing the critical need for accurate corn rust disease identification, this study introduces the innovative Maize-Rust model, an improved YOLOv8 for efficient leaf classification. The model surpasses previous limits in small target detection and lightweight deployment, leveraging advanced technologies including multi-scale BiFPN fusion, lightweight DWConv, and non-parametric attention. On benchmark datasets, it attains a remarkable 94.6% classification accuracy. These advancements hold significant potential for edge computing and real-time applications. Experimental validation confirms the model’s feasibility for rapid, accurate corn disease detection. Furthermore, this study integrates the Maize-Rust model with cloud servers to create a mini-program for non-destructive, efficient, real-time disease monitoring. Future work will concentrate on enhancing disease identification in complex field conditions and optimizing models for improved performance and applicability.

## Data Availability

The raw data supporting the conclusions of this article will be made available by the authors, without undue reservation.

## References

[B1] Ahila Priyadharshini R. Arivazhagan S. Arun M. Mirnalini A. (2019). Maize leaf disease classification using deep convolutional neural networks. Neural Comput. Applic 31, 8887–8895. doi: 10.1007/s00521-019-04228-3

[B2] Aravind K. R. Raja P. Mukesh K. V. Aniirudh R. Ashiwin R. Szczepanski C. (2018). Disease classification in maize crop using bag of features and multiclass support vector machine., in 2018 2nd International Conference on Inventive Systems and Control (ICISC), 1191–1196. doi: 10.1109/ICISC.2018.8398993

[B3] Bebronne R. Carlier A. Meurs R. Leemans V. Vermeulen P. Dumont B.. (2020). In-field proximal sensing of septoria tritici blotch, stripe rust and brown rust in winter wheat by means of reflectance and textural features from multispectral imagery. Biosyst. Eng. 197, 257–269. doi: 10.1016/j.biosystemseng.2020.06.011

[B4] Brewbaker J. L. Kim S. K. So Y. S. Logroño M. Moon H. G. Ming R.. (2011). General resistance in maize to southern rust (Puccinia polysora underw.). Crop Sci. 51, 1393–1409. doi: 10.2135/cropsci2010.06.0327

[B5] Cai J. Pan R. Lin J. Liu J. Zhang L. Wen X.. (2023). Improved EfficientNet for corn disease identification. Front. Plant Sci. 14. doi: 10.3389/fpls.2023.1224385 PMC10519789 37767299

[B6] Chen J. Chen J. Zhang D. Sun Y. Nanehkaran Y. A. (2020). Using deep transfer learning for image-based plant disease identification. Comput. Electron. Agric. 173, 105393. doi: 10.1016/j.compag.2020.105393

[B7] Chen J. Ma A. Huang L. Li H. Zhang H. Huang Y.. (2024). Efficient and lightweight grape and picking point synchronous detection model based on key point detection. Comput. Electron. Agric. 217, 108612. doi: 10.1016/j.compag.2024.108612

[B8] Crouch J. A. Szabo L. J. (2011). Real-Time PCR Detection and Discrimination of the Southern and Common Corn Rust Pathogens *Puccinia polysora* and *Puccinia sorghi* . Plant Dis. 95, 624–632. doi: 10.1094/PDIS-10-10-0745 30731892

[B9] Ferentinos K. P. (2018). Deep learning models for plant disease detection and diagnosis. Comput. Electron. Agric. 145, 311–318. doi: 10.1016/j.compag.2018.01.009

[B10] Garhwal A. S. Pullanagari R. R. Li M. Reis M. M. Archer R. (2020). Hyperspectral imaging for identification of Zebra Chip disease in potatoes. Biosyst. Eng. 197, 306–317. doi: 10.1016/j.biosystemseng.2020.07.005

[B11] Geng Q. Zhang H. Gao M. Qiao H. Xu X. Ma X. (2024). A rapid, low-cost wheat spike grain segmentation and counting system based on deep learning and image processing. Eur. J. Agron. 156, 127158. doi: 10.1016/j.eja.2024.127158

[B12] Guan H. Deng H. Ma X. Zhang T. Zhang Y. Zhu T.. (2024). A corn canopy organs detection method based on improved DBi-YOLOv8 network. Eur. J. Agron. 154, 127076. doi: 10.1016/j.eja.2023.127076

[B13] Hou Q. Zhou D. Feng J. (2021). Coordinate attention for efficient mobile network design. in 2021 IEEE/CVF Conference on Computer Vision and Pattern Recognition (CVPR), 13708–13717. doi: 10.1109/CVPR46437.2021.01350

[B14] Hu J. Shen L. Albanie S. Sun G. Wu E. (2019). Squeeze-and-Excitation Networks, in 2018 IEEE/CVF Conference on Computer Vision and Pattern Recognition, 7132–7141. doi: 10.1109/CVPR.2018.00745

[B15] Kaiser L. Gomez A. N. Chollet F. (2017). Depthwise separable convolutions for neural machine translation. Available online at: http://arxiv.org/abs/1706.03059 (Accessed March 15, 2024).

[B16] Khan F. Zafar N. Tahir M. N. Aqib M. Waheed H. Haroon Z. (2023). A mobile-based system for maize plant leaf disease detection and classification using deep learning. Front. Plant Sci. 14. doi: 10.3389/fpls.2023.1079366 PMC10226393 37255561

[B17] Lin Y. Wang L. Chen T. Liu Y. Zhang L. (2024). Monitoring system for peanut leaf disease based on a lightweight deep learning model. Comput. Electron. Agric. 222, 109055. doi: 10.1016/j.compag.2024.109055

[B18] Liu W. Anguelov D. Erhan D. Szegedy C. Reed S. Fu C.-Y.. (2016). SSD: single shot multiBox detector, in Computer Vision – ECCV 2016, 21–37. doi: 10.1007/978-3-319-46448-0_2

[B19] Liu G. Hu Y. Chen Z. Guo J. Ni P. (2023). Lightweight object detection algorithm for robots with improved YOLOv5. Eng. Appl. Artif. Intell. 123, 106217. doi: 10.1016/j.engappai.2023.106217

[B20] Liu Q. Lv J. Zhang C. (2024). MAE-YOLOv8-based small object detection of green crisp plum in real complex orchard environments. Comput. Electron. Agric. 226, 109458. doi: 10.1016/j.compag.2024.109458

[B21] Lv M. Zhou G. He M. Chen A. Zhang W. Hu Y. (2020). Maize leaf disease identification based on feature enhancement and DMS-robust alexnet. IEEE Access 8, 57952–57966. doi: 10.1109/ACCESS.2020.2982443

[B22] Mei J. Zhou S. Liu W. (2023). Gene-for-gene-mediated resistance to southern corn rust in maize. Trends Plant Sci. 28, 255–258. doi: 10.1016/j.tplants.2022.12.002 36522259

[B23] Meng Z. Du X. Xia J. Ma Z. Zhang T. (2024). Real-time statistical algorithm for cherry tomatoes with different ripeness based on depth information mapping. Comput. Electron. Agric. 220, 108900. doi: 10.1016/j.compag.2024.108900

[B24] Park J. Woo S. Lee J.-Y. Kweon I. S. (2020). A simple and light-weight attention module for convolutional neural networks. Int. J. Comput. Vis. 128, 783–798. doi: 10.1007/s11263-019-01283-0

[B25] Peng Y. Zhao S. Liu J. (2021). Fused-deep-features based grape leaf disease diagnosis. Agronomy 11, 2234. doi: 10.3390/agronomy11112234

[B26] Redmon J. Farhadi A. (2018). YOLOv3: an incremental improvement. arXiv. doi: 10.48550/arXiv.1804.02767

[B27] Reis D. Kupec J. Hong J. Daoudi A. (2024). Real-time flying object detection with YOLOv8. arXiv. doi: 10.48550/arXiv.2305.09972

[B28] Ren S. He K. Girshick R. Sun J. (2017). Faster R-CNN: towards real-time object detection with region proposal networks. IEEE Trans. Pattern Analysis Mach. Intell. 39, 1137–1149. doi: 10.48550/arXiv.1506.01497 27295650

[B29] Sinha R. Khot L. R. Rathnayake A. P. Gao Z. Naidu R. A. (2019). Visible-near infrared spectroradiometry-based detection of grapevine leafroll-associated virus 3 in a red-fruited wine grape cultivar. Comput. Electron. Agric. 162, 165–173. doi: 10.1016/j.compag.2019.04.008

[B30] Sun L. He J. Zhang L. (2024). CASF-MNet: multi-scale network with cross attention mechanism and spatial dimension feature fusion for maize leaf disease detection. Crop Prot. 180, 106667. doi: 10.1016/j.cropro.2024.106667

[B31] Tan M. Pang R. Le Q. V. (2020).EfficientDet: scalable and efficient object detection. Available online at: http://arxiv.org/abs/1911.09070 (Accessed September 6, 2023).

[B32] Waheed A. Goyal M. Gupta D. Khanna A. Hassanien A. E. Pandey H. M. (2020). An optimized dense convolutional neural network model for disease recognition and classification in corn leaf. Comput. Electron. Agric. 175, 105456. doi: 10.1016/j.compag.2020.105456

[B33] Wang C.-Y. Bochkovskiy A. Liao H.-Y. M. (2023). YOLOv7: Trainable bag-of-freebies sets new state-of-the-art for real-time object detectors. in 2023 IEEE / CVF Conference on Computer Vision and Pattern Recognition ( CVPR ), 7464–7475. doi: 10.1109/CVPR52729.2023.00721

[B34] Wang H. Li Y. Minh Dang L. Moon H. (2022). An efficient attention module for instance segmentation network in pest monitoring. Comput. Electron. Agric. 195, 106853. doi: 10.1016/j.compag.2022.106853

[B35] Wang C.-Y. Yeh I.-H. Liao H.-Y. M. (2024). YOLOv9: learning what you want to learn using programmable gradient information. doi: 10.48550/arXiv.2402.13616

[B36] Woo S. Park J. Lee J.-Y. Kweon I. S. (2018). CBAM: Convolutional Block Attention Module. in Computer Vision – ECCV 2018, eds. Ferrari V. Hebert M. Sminchisescu C. Weiss Y. (Cham: Springer International Publishing), 3–19. doi: 10.1007/978-3-030-01234-2_1

[B37] Xie Q. Wu M. Bao J. Zheng P. Liu W. Liu X.. (2023). A deep learning-based detection method for pig body temperature using infrared thermography. Comput. Electron. Agric. 213, 108200. doi: 10.1016/j.compag.2023.108200

[B38] Xu W. Xu T. Alex Thomasson J. Chen W. Karthikeyan R. Tian G.. (2023). A lightweight SSV2-YOLO based model for detection of sugarcane aphids in unstructured natural environments. Comput. Electron. Agric. 211, 107961. doi: 10.1016/j.compag.2023.107961

[B39] Yang M. Kang X. Qiu X. Ma L. Ren H. Huang C.. (2024). Method for early diagnosis of verticillium wilt in cotton based on chlorophyll fluorescence and hyperspectral technology. Comput. Electron. Agric. 216, 108497. doi: 10.1016/j.compag.2023.108497

[B40] Yang G. Yang Y. He Z. Zhang X. He Y. (2022). A rapid, low-cost deep learning system to classify strawberry disease based on cloud service. J. Integr. Agric. 21, 460–473. doi: 10.1016/S2095-3119(21)63604-3

[B41] Yu H. Liu J. Chen C. Heidari A. A. Zhang Q. Chen H.. (2021). Corn leaf diseases diagnosis based on K-means clustering and deep learning. IEEE Access 9, 143824–143835. doi: 10.1109/ACCESS.2021.3120379

[B42] Zeng W. Li H. Hu G. Liang D. (2022). Lightweight dense-scale network (LDSNet) for corn leaf disease identification. Comput. Electron. Agric. 197, 106943. doi: 10.1016/j.compag.2022.106943

[B43] Zhao Y. Sun C. Xu X. Chen J. (2022). RIC-Net: A plant disease classification model based on the fusion of Inception and residual structure and embedded attention mechanism. Comput Electron Agric. 193, 106644. doi: 10.1016/j.compag.2021.106644

[B44] Zhao S. Peng Y. Liu J. Wu S. (2021). Tomato leaf disease diagnosis based on improved convolution neural network by attention module. Agriculture 11, 651. doi: 10.3390/agriculture11070651

